# 2184: Evaluating the association among biological, social, and nutritional status on adolescent pregnancy rates, physiology and birth outcomes using electronic health records data

**DOI:** 10.1017/cts.2017.251

**Published:** 2018-05-10

**Authors:** Amanda Cheng, Caroline S. Jiang, Mireille McLean, Jan L. Breslow, Peter R. Holt, Rhonda G. Kost, Kimberly S. Vasquez, Dena Moftah, Daryl L. Wieland, Peter S. Bernstein, Siobhan Dolan

## Abstract

OBJECTIVES/SPECIFIC AIMS: To build a multisite deidentified database of female adolescents, aged 12–21 years (January 2011–December 2012), and their subsequent offspring through 24 months of age from electronic health records (EHRs) provided by participating Community Health. METHODS/STUDY POPULATION: We created a community-academic partnership that included New York City Community Health Centers (n=4) and Hospitals (n=4), The Rockefeller University, The Sackler Institute for Nutrition Science and Clinical Directors Network (CDN). We used the Community-Engaged Research Navigation model to establish a multisite deidentified database extracted from EHRs of female adolescents aged 12–21 years (January 2011–December 2012) and their offspring through 24 months of age. These patients received their primary care between 2011 and 2015. Clinical data were used to explore possible associations among specific measures. We focused on the preconception, prenatal, postnatal periods, including pediatric visits up to 24 months of age. RESULTS/ANTICIPATED RESULTS: The preliminary analysis included all female adolescents (n=49,292) and a subset of pregnant adolescents with offspring data available (n=2917). Patients were mostly from the Bronx; 43% of all adolescent females were overweight (22%) or obese (21%) and showed higher systolic and diastolic blood pressure, blood glucose levels, hemoglobin A1c, total cholesterol, and triglycerides levels compared with normal-weight adolescent females (*p*<0.05). There was a statistically significant association between the BMI status of mothers and infants’ birth weight, with underweight/normal-weight mothers having more low birth weight (LBW) babies and overweight/obese mothers having more large babies. The odds of having a LBW baby was 0.61 (95% CI: 0.41, 0.89) lower in obese compared with normal-weight adolescent mothers. The risk of having a preterm birth before 37 weeks was found to be neutral in obese compared to normal-weight adolescent mothers (OR=0.81, 95% CI: 0.53, 1.25). Preliminary associations are similar to those reported in the published literature. DISCUSSION/SIGNIFICANCE OF IMPACT: This EHR database uses available measures from routine clinical care as a “rapid assay” to explore potential associations, and may be more useful to detect the presence and direction of associations than the magnitude of effects. This partnership has engaged community clinicians, laboratory and clinical investigators, and funders in study design and analysis, as demonstrated by the collaborative development and testing of hypotheses relevant to service delivery.

